# Apparent diffusion coefficient normalization of normal liver: Will it improve the reproducibility of diffusion-weighted imaging at different MR scanners as a new biomarker?

**DOI:** 10.1097/MD.0000000000005910

**Published:** 2017-01-20

**Authors:** Jie Zhu, Jie Zhang, Jia-Yin Gao, Jin-Ning Li, Da-Wei Yang, Min Chen, Cheng Zhou, Zheng-Han Yang

**Affiliations:** a Department of Radiology, Beijing Hospital, National Center of Gerontology; b Department of Radiology, Beijing Friendship Hospital, Capital Medical University, Beijing, China.

**Keywords:** apparent diffusion coefficient, liver, MR diffusion-weighted imaging, normalization

## Abstract

Apparent diffusion coefficient (ADC) measurement in diffusion-weighted imaging (DWI) has been reported to be a helpful biomarker for detection and characterization of lesion. In view of the importance of ADC measurement reproducibility, the aim of this study was to probe the variability of the healthy hepatic ADC values measured at 3 MR scanners from different vendors and with different field strengths, and to investigate the reproducibility of normalized ADC (nADC) value with the spleen as the reference organ. Thirty enrolled healthy volunteers received DWI with GE 1.5T, Siemens 1.5T, and Philips 3.0T magnetic resonance (MR) systems on liver and spleen (session 1) and were imaged again after 10 to 14 days using only GE 1.5T MR and Philips 3.0T MR systems (session 2). Interscan agreement and reproducibility of ADC measurements of liver and the calculated nADC values (ADC_liver_/ADC_spleen_) were statistically evaluated between 2 sessions. In session 1, ADC and nADC values of liver were evaluated for the scanner-related variability by 2-way analysis of variance and intraclass correlation coefficients (ICCs). Coefficients of variation (CVs) of ADCs and nADCs of liver were calculated for both 1.5 and 3.0-T MR system. Interscan agreement and reproducibility of ADC measurements of liver and related nADCs between 2 sessions were found to be satisfactory with ICC values of 0.773 to 0.905. In session 1, the liver nADCs obtained from different scanners were consistent (*P *= 0.112) without any significant difference in multiple comparison (*P *= 0.117 to >0.99) by using 2-way analysis of variance with post-hoc analysis of Bonferroni method, although the liver ADCs varied significantly (*P* < 0.001). nADCs measured by 3 scanners were in good interscanner agreements with ICCs of 0.685 to 0.776. The mean CV of nADCs of both 1.5T MR scanners (9.6%) was similar to that of 3.0T MR scanner (8.9%). ADCs measured at 3 MR scanners with different field strengths and vendors could not be compared directly. Normalization of ADCs, however, may provide better reproducibility by overcoming these potential issues.

## 1 Introduction

As a widely used MR sequence, diffusion-weighted imaging (DWI) based on Brownian motion of molecular allows tissue characterization by probing tissue microstructural changes, and the measurement is quantified as the apparent diffusion coefficient (ADC). Extracranial application of DWI has gained increasing importance over the past decades and become a routine abdominal imaging protocol, particularly in liver imaging. It has been reported to be helpful for detection and characterization of liver lesion, prediction of treatment response, and assessment of chronic liver diseases.^[1–6]^

ADC measurement might be influenced by multiple factors such as b values, respiration condition, field strength, vendor, and other technical parameters.^[7–12]^ Thus, the lack of standardization of ADC measurement is a critical limitation to this quantitative parameter to be an eligible biomarker. ADC values to characterize abdominal lesions or assess treatment response are always measured at either a 1.5- or a 3.0-T MR scanner from different vendors among respective hospitals or for a patient who takes serial follow-up scanning in 1 hospital. Therefore, finding another parameter to minimize these differences and to obtain better reproducibility of ADC values would become beneficial and significant in multicenter studies or serial trials.

Studies initially involving brain followed by abdomen demonstrated that normalized ADC (nADC) with a reference organ reduced the variability of lesion ADCs to improve DWI characterization of pathologic conditions, such as pancreatic cancer and massforming pancreatitis discrimination,^[13]^ aggressiveness of prostate cancer detection,^[14,15]^ and metastatic lymph nodes in cervical cancer characterization.^[16]^ Regarding to the hepatic lesions, Do et al^[17]^ demonstrated that nADC improved diagnostic accuracy for detection of liver fibrosis and cirrhosis compared to absolute ADCs. However, this methodology has not been well verified in minimizing the variety of data caused by scanning techniques and protocols, field strengths, and vendors for different MR scanners.

The purpose of this prospective study was to investigate how the changes of MR scanners of 1.5 and 3.0T with different manufactures affect the liver ADCs in healthy volunteers, and to demonstrate nADC values of liver (using the spleen as reference organ) improved the reproducibility.

## 2 Methods

### 2.1 Ethics statement

This study was approved by the local Institutional Review Board. All patients provided written informed consent before the study and consent to publish the medical images included in the figures.

### 2.2 Study participants

#### 2.2.1 Session 1

During a 4-month clinical trial, 30 healthy adult volunteers (15 men and 15 women; mean age, 26 years; age range, 22–40 years) were recruited into the prospective study to undergo 3 DWI examinations in the upper abdomen with 3 different MR scanners (Siemens 1.5T (Siemens Magnetom Espree [Siemens Healthcare; Erlangen, Germany] with 30 mT/m maximum gradient strength and 100 mT/m/s gradient slew rate), GE 1.5T (GE Signa HD Twin-Speed [GE Medical systems; Milwaukee, WI] with 23/40 mT/m maximum gradient strength and 80/150 mT/m/s gradient slew rate), and Philips 3.0T (Philips Achieva Dual [Philips Medical Systems; Best, The Netherlands] with 30/60 mT/m maximum gradient strength and 200/100 mT/m/s gradient slew rate) MR system). All volunteers had no prior liver/spleen disease history or positive liver/spleen imaging findings other than hemangioma or cyst. To maintain a similar hydration level, the volunteers refrained from eating 4 hours before imaging and were instructed to drink 1 L of water 2 hours before imaging. To make sure that the functional status of the liver and the spleen were similar, all the examinations of each volunteer on 3 different instruments were performed in the resting state on the same day at regular time intervals of 1 to 2 hours.

#### 2.2.2 Session 2

After 10 to 14 days, all of the 30 volunteers were imaged again using 2 of the 3 scanners (GE 1.5T MR and Philips 3.0T MR) with the same protocol as in session 1.

### 2.3 MR imaging (MRI)

A GE 1.5T, a Siemens 1.5T, and a Philips 3.0T magnetic resonance systems were utilized in the study. Each participant received MRI examinations by each of the 3 or 2 instruments on the same day in sessions 1 and 2.

The routine MRI protocol included a transverse breath-hold T1-weighted gradient-recalled echo with in-phase and out-of-phase sequences and a respiratory-triggered T2-weighted fast spin-echo sequence with fat saturation. The DWI parameters of each MR scanner are shown in Table 1.

**Table 1:**
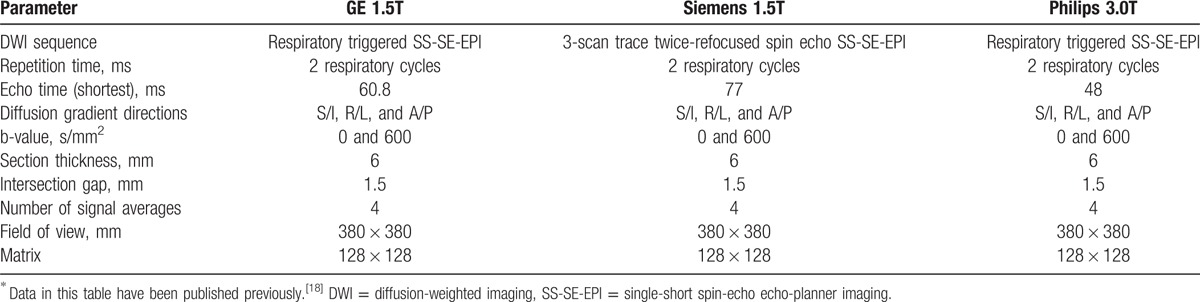
Acquisition^∗^ parameters of DWI at 3 MR instruments.

### 2.4 Quantitative MR image analysis

**Figure 1:**
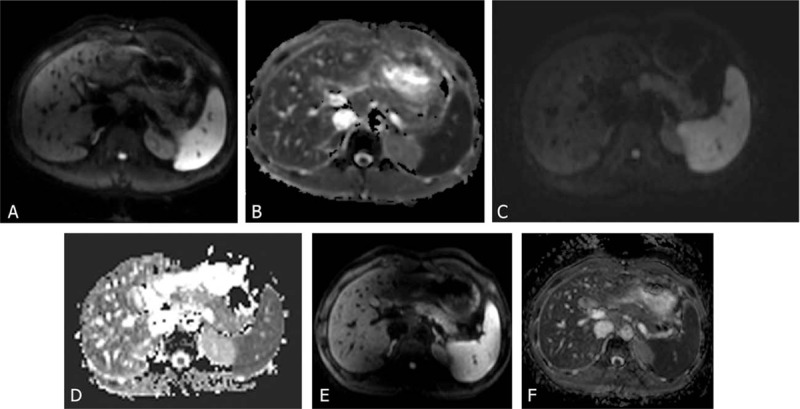
(A–F) Diffusion-weighted images acquired at GE 1.5T (A and B), Siemens 1.5T (C and D), and Philips 3.0T (E and F) in a 34-year-old healthy male volunteer with b values of 600 s/mm^2^ (A, C, and E), and the corresponding apparent diffusion coefficient maps (B, D, and F).

Quantitative analysis was performed on the individual commercial workstations corresponding to each MR scanner. The ADC values of each DWIs were automatically calculated and displayed on corresponding ADC maps. Four nonoverlapping oval or circular regions of interest (ROIs) of 90–110 mm^2^ were drawn on DWIs with b value of 0 s/mm^2^ on the posterior right hepatic lobe and the spleen at the same or interfacing slice. The ROIs were carefully placed in homogenous artifact-free areas with large blood vessels excluded. The ADC values of 4 ROIs on liver and spleen parenchyma were averaged separately to represent the ADCs of each organ in a single individual and MR system. The ROIs were placed as consistently as possible for each scan (Fig. 1). nADC of liver was defined as the ratio of ADC value of the liver parenchyma to spleen ADC measurement.

### 2.5 Statistical analysis

ADCs presented as mean ± standard deviation (SD) were tested first with the Kolmogorov–Smirnov test for normality and then with the Levene test for variance homogeneity.

For GE 1.5T and Philip 3.0 T, interscan agreements of ADC and nADC measurements between sessions 1 and 2 were evaluated by using intraclass correlation coefficients (ICCs) (0–0.20, poor correlation; 0.21–0.40, fair correlation; 0.41–0.60, moderate correlation; 0.61–0.80, good correlation; and 0.81–1.00, excellent correlation). The short-term reproducibility of ADC and nADC measurements was evaluated with the Bland–Altman method.^[19]^ The mean absolute difference (bias) and the 95% confidence interval of the mean difference (limits of agreement [LOA]) between the first and second DWI series were calculated.

Next, in session 1, agreement of ADC measurement of liver/spleen and the related nADCs at 3 MR scanners was analyzed by calculating ICCs. All of the data variations were assessed by using 2-way analysis of variance, with a post-hoc analysis of Bonferroni method performed to adjust for multiple comparisons.

Finally, coefficients of variations (CV = SD divided by mean) were calculated overall across the 3 MR scanners of session 1 while they were also calculated separately for MR scanners with field strengths of 1.5T (both 1.5T MR data were combined to obtain the overall CVs for 1.5T MR scanners) and 3.0T.

Statistical analysis was performed by using SPSS (version 17.0; Chicago, IL) and MedCalc (version 16.2; Mariakerke, Belgium) software. A *P* value of less than 0.05 was considered as a significant difference.

## 3 Results

All imaging data of 30 volunteers were adopted in our research without affection of artifact.

### 3.1 Interscan agreement and reproducibility of ADC and nADC measurement between 2 sessions

**Figure 2:**
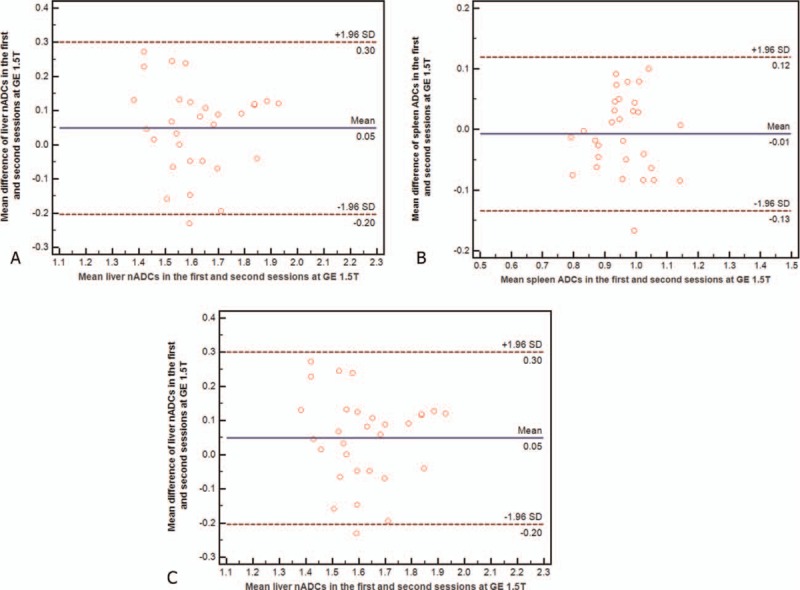
(A–C) Bland–Altman plots show reproducibility of apparent diffusion coefficients (ADCs) (×10^−3^ mm^2^/s) of liver (A), spleen (B), and normalized ADC (C) between first and second sessions at GE 1.5T. Blue line = mean absolute difference, red lines = 95% confidence interval of the mean difference (limit of agreement).

**Figure 3:**
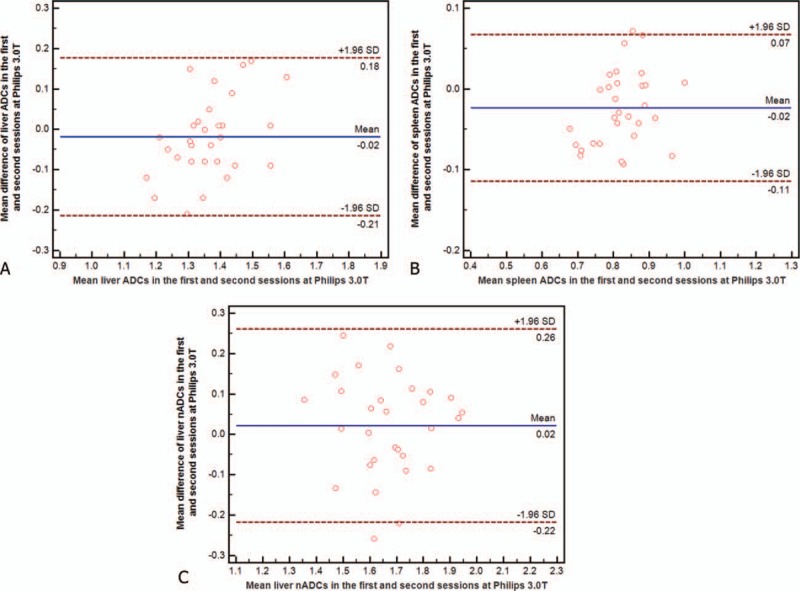
(A–C) Bland–Altman plots show reproducibility of apparent diffusion coefficients (ADCs) (×10^−3^ mm^2^/s) of liver (A), spleen (B), and normalized ADC (C) between first and second sessions at Philips 3.0T. Blue line = mean absolute difference, red lines = 95% confidence interval of the mean difference (limit of agreement).

The interscan agreements between 2 imaging sessions of ADC measurement of liver and spleen, as well as nADCs at GE 1.5T and Philip 3.0T, were satisfactory with ICCs ranged from 0.773 to 0.905, indicating a good-to-excellent correlation (Table 2). Bland–Altman reproducibility analysis of ADC and nADC measurements showed that 96.7% (29/30) of the ADC/nADC bias was inside the LOA in terms of the mean ADC values of liver/spleen and nADCs at both GE 1.5T (Fig. 2) and Philips 3.0T (Fig. 3). The ranges of mean differences of ADCs (nADC) ± LOA between 2 imaging sessions were (0.035 ± 0.152) × 10^−3^ mm^2^/s (liver), (−0.007 ± 0.127) × 10^−3^ mm^2^/s (spleen), and 0.049 ± 0.252 (nADC) at GE1.5T, (−0.018 ± 0.195) × 10^−3^ mm^2^/s (liver), (−0.023 ± 0.091) × 10^−3^ mm^2^/s (spleen), and 0.022 ± 0.239) (nADC) at Philips 3.0T.

**Table 2:**
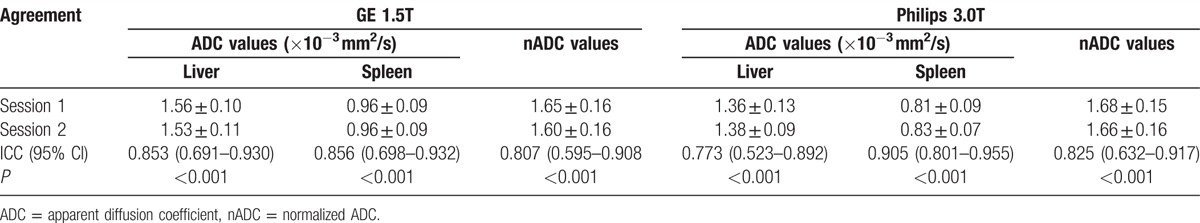
Interscan agreement (between sessions 1 and 2, with a time interval of 10–14 days) for ADC measurements (×10^−3^ mm^2^/s) of liver/spleen and nADCs at GE 1.5T and Philips 3.0T, respectively.

### 3.2 Reproducibility of ADCs and nADCs of liver at 3 MR scanners

In session 1, the mean ADCs of liver measured by different scanners were not consistent with significant differences found among these 3 MR scanners (*P* < 0.001) (Table 3) and between any 2 of them (*P* = 0.001 for GE 1.5T vs Siemens 1.5T, *P* < 0.001 for GE 1.5T vs Philips 3.0T, and Siemens 1.5T vs Philips 3.0T). The mean ADCs of spleen measured by 3 MR scanners shared the same results with ADCs of liver, except that no significant difference was found between GE 1.5T and Siemens 1.5T (*P* = 0.437).

**Table 3:**
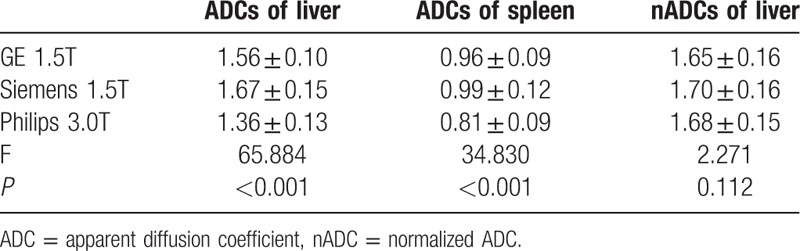
Comparison of ADCs (×10^−3^ mm^2^/s) of liver, spleen parenchyma, and normalized ADCs of liver (nADCs = ADCliver/ADCspleen) among 3 MR scanners in session 1.

In contrast, nADCs of liver were very close to each other without significant difference among the MR scanners (*P* = 0.112) (Table 3) and between any 2 of them (*P* = 0.117 for GE 1.5T vs Siemens 1.5T, *P* = 0.585 for GE 1.5T vs Philips 3.0vT, and *P* > 0.99 for Siemens 1.5T vs Philips 3.0T).

nADCs of liver measured by 3 MR scanners were in good interscanner agreements with ICCs of 0.685 to 0.776, while ADCs of liver and spleen among scanners showed poor-to-moderate agreements with ICCs lower than 0.6 (Table 4).

**Table 4:**
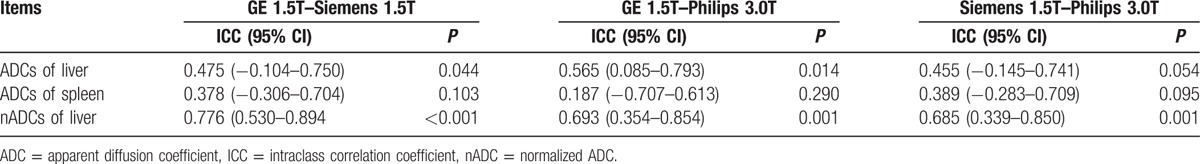
ICCs of ADC measurement of liver, spleen, and relative nADCs at 3 MR scanners in session 1.

### 3.3 CVs of ADCs and nADCs of liver

Regarding with the measured results of CVs, nADCs of liver showed slightly higher CVs (9.7% for GE 1.5T, 9.4% for Siemens 1.5T, and 8.9% for Philips 3.0T) than ADCs (6.4%, 9%, and 9.6% for 3 scanners respectively), but all of the CVs were within the previously reported data range.^[10,12,20]^ After integrating data of both 1.5T MR scanners to calculate CV of nADCs at 2 1.5T MR scanners, the results showed that CV of nADCs at 1.5T MR scanners was 9.6%, close to that at 3.0T MR scanner (8.9%).

## 4 Discussion

Our results demonstrated that liver nADCs were not affected by changes of MR scanners with different field strengths and vendors (*P* = 0.112 within scanners, *P* = 0.117 to >0.99 for multiple comparison between any 2 of 3 MR scanners) compared to liver ADCs. Good correlations between any 2 different MR instruments were observed (ICCs were 0.685–0.776). The CVs of nADCs at 3 MR scanners were in the acceptable range of clinical application. The preliminary research by Donati et al^[12]^ found that CVs of liver ADCs at 3.0 T were markedly higher than those at 1.5 T. In our research, nADCs on 1.5 T and 3.0 T MR scanners had the negligible CV difference (9.6% for 1.5 T MR scanners and 8.9% for 3.0 T MR scanner), indicating that the variability of nADCs were not affected by MR instruments with the different field strengths. So, nADCs were stable and can be applied as a quantification tool for DWI of the liver performed at MR scanners with different field strengths and vendors.

So far there has been no full consensus on which reference organ is the most appropriate and how to define the nADC. Researchers using nADC to characterize diseases often define it as ADC_lesion_/ADC_reference organ_. Spleen, muscle, cortex of kidney, adrenal gland, and adjacent healthy parenchyma had already been reported to be the reference organs in the ADC measurement of abdomen.^[14,16–17,20]^ Spleen is chosen more frequently as it is usually less affected by body habitus, fatty infiltration, aging, and gender.^[21,22]^ In our research on normal liver parenchyma, similar way was used to define nADC as ADC_liver_/ADC_reference organ_. Considering more reliable calculative approaches can be identified, our future research should be focused on how to sufficiently characterize hepatic parenchyma or lesions by using nADC.

Our study showed that the interscan agreement and short-term reproducibility of liver and spleen ADCs, as well as related nADCs were satisfactory. Between 2 imaging sessions (with 10–14 days interval) at both GE 1.5T and Philips 3.0T, the ICCs were 0.773 to 0.905 (*P* < 0.001), and the mean differences of ADCs and nADCs were (0.007–0.035) × 10^−3^ mm^2^/s and 0.022 to 0.049, respectively. Recent studies from other researchers also have verified that DWI has good interscan reproducibility in phantom,^[23,24]^ normal liver,^[20,23]^ and hepatic lesions^[8]^ with excellent interreader agreement.^[12]^ So, ADC and nADC measurement can serve as a reliable quantitative tool over time.

Absolute ADC values measured from different MR scanners are not always consistent and may not be compared directly.^[25–28]^ This issue, however, was addressed by very few researchers of abdomen until the study by Donati et al.^[12]^ They measured the ADC values of upper abdomen at 3 manufactures (GE, Philips, and Siemens) of both 1.5 and 3.0 T. The results showed that there were significant vendor-dependent differences between ADC values in 2 of 7 upper abdominal regions (left and right liver lobes) at 1.5 T and in 4 of 7 upper abdominal regions (left liver lobe, pancreas, and renal cortex and medulla) at 3.0 T. Ye et al^[18]^ found no variance in pancreatic ADCs between 2 MR scanners (GE and Siemens) of 1.5 T. Our results from GE 1.5T and Siemens 1.5T confirmed that the variance of ADC values was only linked to liver (*P* = 0.01), rather than spleen. It seemed that the intervendor differences of ADC measurements were inconsistent in the organ of upper abdomen, and the liver exhibited greater variation than others. For extraabdominal organ, the intervendor differences of ADC measurements were also tested in breast and neck.^[27,28]^ Reason for vendor-related differences of measured ADC values still remains unknown. We speculated that it could be attributed to the hardware and software related issues, such as field inhomogeneity, methodological, coil systems, and intrinsic physical factors related to the differences in the design of the DWI sequences.^[25,29]^

In our research, ADCs of liver at Philips 3.0T were lower than those at GE 1.5T and Siemens 1.5T. It should be noticed that our MR scanners with different field strengths were from different vendors. Contradicting results of how ADCs of the liver parenchyma,^[10,12,30]^ some other organs of the abdomen, and the brain^[25,31,32]^ were affected by different field strengths have been reported in literatures. For instance, Dale et al^[30]^ found that hepatic ADCs at 3.0T MR were higher than those at 1.5T MR and assumed that the noise floor issues from lower signal-to-noise ratio (SNR) reduced ADC values. Rosenkrantz et al^[10]^ did not observe the field-strength-related difference, and they thought that the T2 properties offset the potential increase in SNR at 3.0 T to mitigate the impact of noise floor at 1.5 T as T2 relaxation time of the liver decreased with the magnetic field strength. We assumed that small number (10–16 cases) in these prior studies might partially lead to this contradiction. We also noticed that some effects attributed to ADC calculation, such as echo time, diffusion time, amplitude, and separation of diffusion gradient were not easy to be strictly uniformed when detecting the impact of field strength on ADC measurements.^[31,33]^ As ADC value was affected by SNR of high b values in liver, it is important for 3.0T MR scanners to have high SNR with improved imaging techniques and reasonable diffusion-weighted parameters to get precise ADC values.^[11,32]^

The following limitations should be acknowledged in our study: first, the volunteers were relatively young and middle-aged without covering different age brackets. Elderly volunteers might be less cooperative in controlling their breathing for RT DWI. Second, MR scanners of 1.5 and 3.0 T were from different vendors for assessing the effect of field strengths on ADC measurement. It was hard to tease out the contribution of 2 factors. Third, only 2 b values (0 and 600 s/mm^2^) were adapted for ADC measurement in this study due to the widely accepted concept in clinical trials that 2 b values, 0 s/mm^2^ and a higher 1 (≥500 s/mm^2^), are sufficient to characterize liver lesions. Theoretically, ADCs obtained by the intravoxel incoherent motion (IVIM) model with multiple b values could provide more precise information on tissue perfusion and diffusivity base.^[34]^ Nevertheless, multiple b values generally translated to longer acquisition time, which might impact on the clinical workflow. Further studies on the reproducibility of IVIM should be considered. Another limitation was that the dedicated workstations were chosen instead of uniform software platform. Vendor-independent software is preferred in the present ADC measurement associated research, but the workstation is valid in normal clinical practice. Several researches focusing on the affection of software on ADC measurement of phantom and liver lesions demonstrated that no significant difference of ADC values among dedicated workstations, main MRI consoles, and PACS systems.^[25,35]^

In conclusion, ADC values of liver and spleen showed good reproducibility measured at the same MR scanner over a short period of 10 to 14 days, but we observed substantial variability of liver ADC at different MR scanners with different vendors and field strengths. Our research had demonstrated that to overcome these adverse effects caused by the uncertainty of various scanners, nADC value with spleen as a reference organ could be used as a reliable parameter to quantitatively characterize liver microstructural changes.
